# Biological Properties of Milk-Derived Extracellular Vesicles and Their Physiological Functions in Infant

**DOI:** 10.3389/fcell.2021.693534

**Published:** 2021-06-25

**Authors:** Xue Jiang, Lianghui You, Zhenxing Zhang, Xianwei Cui, Hong Zhong, Xingzhen Sun, Chenbo Ji, Xia Chi

**Affiliations:** ^1^Women’s Hospital of Nanjing Medical University, Nanjing Maternity and Child Health Care Hospital, Nanjing, China; ^2^The Affiliated Huaian No.1 People’s Hospital of Nanjing Medical University, Huaian, China; ^3^The First Affiliated Hospital With Nanjing Medical University, Nanjing, China

**Keywords:** milk, microRNA, extracellular vesicles, exosome, infant, growth and development

## Abstract

Extracellular vesicles (EVs) are released by all cells under pathological and physiological conditions. EVs harbor various biomolecules, including protein, lipid, non-coding RNA, messenger RNA, and DNA. In 2007, mRNA and microRNA (miRNA) carried by EVs were found to have regulatory functions in recipient cells. The biological function of EVs has since then increasingly drawn interest. Breast milk, as the most important nutritional source for infants, contains EVs in large quantities. An increasing number of studies have provided the basis for the hypothesis associated with information transmission between mothers and infants via breast milk-derived EVs. Most studies on milk-derived EVs currently focus on miRNAs. Milk-derived EVs contain diverse miRNAs, which remain stable both *in vivo* and *in vitro*; as such, they can be absorbed across different species. Further studies have confirmed that miRNAs derived from milk-derived EVs can resist the acidic environment and enzymatic hydrolysis of the digestive tract; moreover, they can be absorbed by intestinal cells in infants to perform physiological functions. miRNAs derived from milk EVs have been reported in the maturation of immune cells, regulation of immune response, formation of neuronal synapses, and development of metabolic diseases such as obesity and diabetes. This article reviews current status and advances in milk-derived EVs, including their history, biogenesis, molecular contents, and biological functions. The effects of milk-derived EVs on growth and development in both infants and adults were emphasized. Finally, the potential application and future challenges of milk-derived EVs were discussed, providing comprehensive understanding and new insight into milk-derived EVs.

## Background

The Developmental Origins of Health and Disease hypothesis, also known as DoHad hypothesis, indicates that early life is the origin of health and disease in adulthood (Barker, 2007). Infancy is the window of human life, and breast milk is the most ideal source of nutrition during this period. Breastfeeding has immediate and long-term health effects on newborns, children, and even adults (Victora et al., 2016). The World Health Organization recommends exclusive breastfeeding for the first 6 months of life and continued breastfeeding until the age of two or more years. The benefits of breastfeeding are associated with the balanced supply of nutrients and multiple bioactive components in breast milk. Thus, the components of breast milk deserved to be recognized and clarified.

Breast milk is not only rich in various nutrients, including proteins, fats, carbohydrates, minerals, and vitamins, which can provide the energy necessary for growth and development in infancy; breast milk is also rich in biological ingredients, including lactoferrin, immunoglobulin, growth factors, oligosaccharides, and polyunsaturated fatty acids, among others (Victora et al., 2016). These ingredients affect the development of the gastrointestinal tract, brain, immune system, and so on in infants. The composition of breast milk is not constant and is related to race, heredity, nutritional status, and gestational age, etc. It changes with the lactation period to adapt to the needs of infant growth at different stages. Research efforts have been exerted toward exploring the composition and function of breast milk. However, owing to the complexity of the composition of breast milk, no formula that can perfectly simulate and replace breast milk has thus far been established. Thus, exploring the ingredients of breast milk not only emphasizes the importance of breastfeeding for health in children but also provides a scientific basis for the modification of formula milk.

Extracellular vesicles (EVs) are bilayer membrane vesicles released from cells into the extracellular space. Almost every living organism, including all types of cells in animals, plants, and microorganisms, can release exosomes. As an important pathway for intercellular signal transmission, EVs carry proteins, lipids, and nucleic acids (DNA, microRNA, and messenger RNA), which come from the original cell.

EVs used to be regarded as cell membrane fragments for cell waste discharge (Wolf, 1967). Further research has shown that EVs released from the original cells bind to specific receptor cells and are thus involved in multiple life processes, such as angiogenesis, inflammation, immune recognition and response, and neuron signaling. Mammalian milk is rich in exosomes (Admyre et al., 2007). Similar to other body fluids, milk-derived EVs are composed of a variety of RNA, lipids, proteins, and so on. The bilayer membrane structure of milk-derived EVs makes it possible to survive gastric/pancreatic digestion, can protect the contents from being destroyed by the digestion of stomach and pancreas and thus can further be absorbed by intestinal cells and play biological activities (Liao et al., 2017). Milk-derived EVs have been proved to participate in the regulation of physiological and pathological processes in infants, including immunity, neural development, and metabolic regulation (Zempleni et al., 2017). However, the mechanism allowing milk-derived EVs to transfer genetic messages to infants and further affect gene expression and regulation of cellular events has yet to be explored and clarified.

The current study discusses research progress in milk-derived EVs in recent years and focuses on its major content, microRNA (miRNA), to demonstrate the potential mechanism underlying the absorption of milk EVs derived miRNAs, their transfer into the blood circulation to target organs or cells, and their regulation of gene expression and physiological activities in target cells. The effects of miRNAs from milk-derived EVs on growth and development in infants were underscored. Potential applications of milk-derived EVs in therapeutic approaches, future prospects, and challenges to the application of EVs were also reviewed. This information is expected to elucidate the biological functions of milk-derived EVs.

## Introduction of Evs

### History, Definition, and Biogenesis of EVs

Small vesicles released by the fusion of multivesicular bodies and plasma membrane in sheep reticulum were observed in 1983 by Johnstone (Pan and Johnstone, 1983), who referred to these microvesicles as “exosomes” in a 1987 study (Johnstone et al., 1987). Exosomes were initially regarded to represent cellular debris and metabolic waste. Raposo et al. (1996) found that EVs derived from human and mouse B lymphocytes could participate in the cellular immune response. Valadi et al. (2007) demonstrated that exosomes could carry messenger RNA (mRNA) and miRNA and deliver information between cells. This finding prompted an upsurge in research on the biological functions of exosomes, particularly its cargo miRNA.

EVs are small vesicles secreted by original cells into the extracellular space and carry various biological factors including proteins, DNAs, RNAs, lipids, and other contents from the original cells. The classification of EVs is always evolving, but they can be roughly divided, based on size and biogenesis, into two categories, exosomes and ectosomes (Cocucci and Meldolesi, 2015; Thery et al., 2018).

Exosomes, which are formed by endocytosis, have a diameter of 40–160 nm (an average of 100 nm). Exosome biogenesis occurs as follows: cell membranes sag to form early endosomes, and early endosomes are further invaginated to form multivesicular bodies (MVBs). Some parts of MVBs are then integrated with lysosomes and then degraded, whereas the other parts fuse with the plasma membrane to release internal vesicles, referred to as exosomes (Colombo et al., 2014). By contrast, ectosomes mainly contain microvesicles, microparticles, and large vesicles in the size varies from ∼50 nm to 1 μm in diameter. They are vesicles generated through direct outward budding of the plasma membrane (Thery et al., 2018). In this review, we mainly focus on exosomes and other EVs in milk are also discussed when relevant. Assigning an EV to a biogenesis pathway remains difficult unless researchers can confirm the subcellular origin of EVs. Therefore, some of their findings may reflect exosomes mixed with other kinds of EVs (Kalluri and LeBleu, 2020).

**FIGURE 1:**
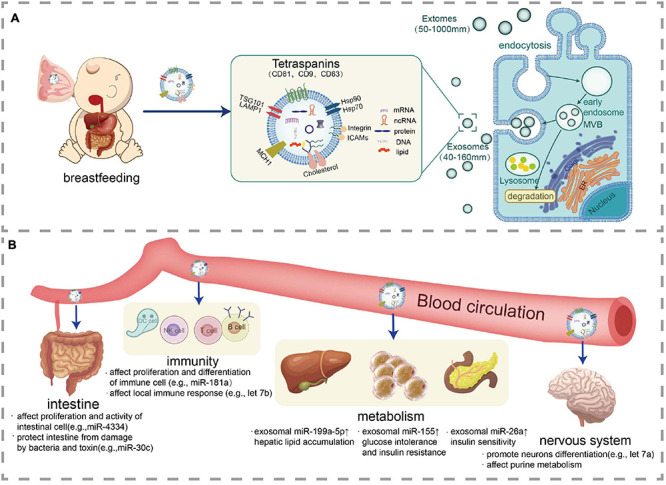
Synthesis, secretion, transport, and effects of milk-derived EVs. **(A)** Representation of EVs production from cells including milk and an illustration of EVs especially exosomes structure with their cargos including nucleic acid (mRNA, ncRNA, and DNA), protein, and lipids. **(B)** EVs are absorbed by the intestinal cells of the infant and are transported to various tissues of the body via blood circulation to exert biological activities including immune regulation, metabolic regulation, and neural development.

Almost every living organism, including animals, plants, and microorganisms, releases exosomes or exosome-like vesicles (Kalluri and LeBleu, 2020). Exosomes exist in blood, urine, breast milk, saliva, semen, cerebrospinal fluid, amniotic fluid, bronchoalveolar lavage fluid, pleural effusion, and other body fluids. Figure 1A briefly depicted the synthesis, secretion, and transport of milk-derived EVs.

### Components of EVs

EVs are highly heterogeneous due to the differences of their size, components, functional impact on recipient cells, and cell of origin. EVs mainly consist of cellular origin lipids, proteins, and nucleic acids (RNA and DNA). The composition of EVs depends on the type and physiological state of the original cells; however, all EVs contain similar conserved proteins, including membrane transport and fusion proteins, tetraspanins (CD63, CD9, CD81), major histocompatibility complex class I and II molecules (MHC I/II), cytosolic proteins such as heat shock proteins (HSPs), and proteins involved in multivesicular body biogenesis, such as Alix and TSG101 (Kalluri and LeBleu, 2020). These proteins are often used as “markers” in EVs characterization. EVs also carry genetic material such as RNA and DNA from original cells (Takahashi et al., 2017). Exosomal RNA cargo is diverse, including coding RNA (mRNA) and non-coding RNA (ncRNA), which mainly include miRNA, transfer RNA, ribosomal RNA, small nucleolar RNA, circular RNA (circRNA), and long non-coding RNA (lncRNA). Updated data from the ExoCarta database reveal that 9769 proteins, 3408 mRNAs, 2838 miRNAs, and 1116 lipids have been identified in exosomes from different cellular sources^1^ (updated in 2016).

### Biological Functions and Regulatory Mechanisms of EVs

EVs have been demonstrated to play an important role in various biological processes, such as angiogenesis, antigen presentation, cell homeostasis and apoptosis, inflammation, and neurodegeneration (Kalluri and LeBleu, 2020). These effects are attributed to the ability of EVs to transfer RNAs, proteins, and lipids, thereby affecting the physiological and pathological processes of various diseases, including cancer, neurodegenerative diseases, infections, and autoimmune diseases.

Further research reveals how EVs cargos function individually: (1) **mRNA**. In, Valadi et al. (2007) reported that exosomes secreted by mouse mast cells could be captured by human mast cells, and mRNAs in exosomes could enter the cytoplasm and be translated into proteins. (2) **miRNA:** miRNAs transferred via EVs enter the target cell and regulate gene expression at the post-transcriptional level (Kosaka et al., 2010a; Pegtel et al., 2010; Zhang et al., 2010). (3) **lncRNA:** As a transport medium, EV encapsulates lncRNA, aiding the penetration of target cells and tissues and performing its function in epigenetic regulation, competitive binding miRNA, pre-transcriptional and post-transcriptional regulation (Mohammad et al., 2010; Mi et al., 2020). (4) **circRNA:** Exosomal circPACRGL can promote the expression of transforming growth factor macrophage by activating miR-142-3p/miR-506-3p (Shang et al., 2020). (5) **protein:** Chalmin et al. (2010) found that the tumor-derived exosomal protein Hsp72 restrained tumor immune surveillance by promoting the suppressive functions of myeloid-derived suppressor cells. (6) **lipid:** Exosomes contain bioactive lipids transported between cells, which can promote inflammatory diseases (Esser et al., 2010) and immunosuppression (Salimu et al., 2017).

## Evs in Breast Milk

### The Possible Source of EVs in Breast Milk

Admyle (Admyre et al., 2007) first reported in 2007 that human breast milk is rich in exosomes, but the origin of these exosomes remains uncertain. They may originate from breast epithelial cells (Modepalli et al., 2014; Larssen et al., 2017), macrophages, lymphocytes, or even cells from other parts of the body, which reach breast milk through the blood circulation.

### Components of EVs in Breast Milk

Similar to blood, urine, and other body fluids, human breast milk-derived EVs are mainly composed of lipids, proteins, RNA, and DNA released by cells. **RNA:** Milk-derived EVs contain coding RNA (which is mRNA) and non-coding RNA. More than 19,000 and 16,000 mRNAs have thus far been detected in bovine and porcine milk-derived exosomes (Izumi et al., 2015; Chen et al., 2017); however, to the best of our knowledge, no relevant data exist in human milk exosomal mRNAs. Non-coding RNA is predominantly miRNA, which comprises 15% of the average milk-derived exosomal RNA content (Sun et al., 2015). More than 1,900 and 800 miRNAs have been detected in EVs derived from human and bovine milk, respectively (Chen et al., 2010; Zempleni et al., 2017). In addition to miRNAs, other non-coding RNAs also exist in milk-derived EVs, including lncRNA, circular RNA, ribosomal RNA, transfer RNA, small nucleolar RNA, and so on. Zeng et al. (2019) identified 3475 novel lncRNAs in bovine milk-derived exosomes by RNA sequencing. These lncRNAs are involved in the regulation of the immune function, osteoblasts, neurodevelopment, and reproduction. LncRNAs in human milk-derived EVs also perform functions related to growth in children and the development of diseases, including allergic diseases, asthma, obesity, and autoimmune diseases (Karlsson et al., 2016). Moreover, circRNAs exist in exosomes derived from bovine (Wang Y. et al., 2019; Ma et al., 2021) and porcine milk (Zeng et al., 2020). A total of 2059 distinct circRNAs from bovine milk-derived exosomes were identified via high-throughput RNA sequencing (Wang Y. et al., 2019). A small quantity of ribosomal RNA was also detected in EVs derived from human and porcine milk (Karlsson et al., 2016; Ma et al., 2018). **Protein and peptide:** Exosomes derived from breast milk are richer in proteins than those derived from other body fluids. Up to 1963, 2107, and 639 proteins have thus far been detected in EVs from human, bovine, and porcine milk, respectively (Reinhardt et al., 2012; van Herwijnen et al., 2016; Chen et al., 2017). Yang et al. (2017) identified 920 exosomal proteins from human and bovine colostrum and mature milk, 575 of which are differentially expressed. Using LC-MS/MS proteomic analysis, a total of 1963 proteins were identified in human milk-derived EVs (van Herwijnen et al., 2016). Remarkably, 633 proteins identified in milk-derived EV have not yet been identified in human milk. These novel proteins were involved in regulation of and inflammation and cell growth, indicating that milk-derived EVs could affect infant’s immune system and developing gastrointestinal tract. Wang X. et al. (2019) showed that 70 exosomal peptides were significantly different between breast milk given to term and premature infants, and these changing peptides are involved in regulating intestinal epithelial cell function. However, proteomics analysis of EVs derived from human milk is still in the primary stages, and the mechanism allowing EVs-derived proteins to exert biological functions has yet to be determined. **Lipids and DNA:** Recently, Chen et al. (2021) identified totally 395 lipids in term and preterm human milk-derived exosomes and top 50 lipids are involved in the regulation of intestinal epithelial cell function. No relevant studies on DNA in milk-derived EVs have thus far been reported. More studies have to be conducted in the future.

### Factors Influencing the Content and Biological Activity of Milk-Derived EVs

Expression of milk-derived EVs is affected by infant and maternal factors. (1) **gestational age:** Gestational age is associated with the content and biological activity of milk-derived EVs. Wang X. et al. (2019) reported that milk-derived exosomes of mothers who delivered preterm infants could more efficiently promote the proliferation of intestinal cells, compared with those of mothers who delivered term infants. They also identified 70 peptides that were differentially expressed in milk-derived exosomes of mothers who delivered preterm and term infants. Carney et al. (2017) indicated that preterm birth also changed the expression of milk-derived exosomal miRNAs and identified 113 exosomal miRNAs that were differentially expressed between preterm and term breast milk. These differential miRNAs were involved in the biosynthesis, catabolism, symbiosis, and handling of the relevant virus, indicating that premature changes the composition of milk exosomal miRNAs to adapt to the special growth pattern of preterm infants. (2) **lactation period:** The concentration and contents of milk-derived EVs change as the lactation period is extended. Exosomes were found to be more highly concentrated in colostrum than in mature milk (Torregrosa Paredes et al., 2014; Gao et al., 2019). Immune-related miRNAs in EVs from colostrum were markedly higher than those from mature milk (Sun et al., 2013), whereas tissue-specific miRNAs such as miR-142-5p (hematopoietic system), miR-122 (liver), and miR-216 (pancreas) were more highly expressed in mature milk, which was consistent with the requirements for infant growth and development at different stages. Consistent with this, panda EVs of colostrum contained miRNAs more closely associated with immune regulation, while those of mature milk were more involved in tissue development and metabolism (Ma et al., 2017). Expression of bovine milk-derived EVs also changed with lactation. For instance, the expression levels of LNC_001182 and LNC_002303 were higher in colostrum than in mature milk exosomes; meanwhile, the expression level of LNC_001442 was higher in the middle and late stages of lactation (Days 150 and 270, respectively) than in colostrum and early stages of lactation (Day 30) (Zeng et al., 2019). Exosomal proteins in colostrum and mature milk were also differentially expressed (Yang et al., 2017). (3) **maternal diet or nutrition:** Animal studies showed that diet changed the expression of miRNAs in milk-derived EVs. Adding ginseng polysaccharides as immunomodulators to the diet of sows altered the expression of immune-related miRNAs in porcine milk-derived exosomes, influencing offspring development (Sun et al., 2019). Quan et al. (2020) replaced a portion of original alfalfa hay in the feed of dairy cows with whole cottonseeds and soybean hulls. They then found that the concentration of milk-derived EVs in dairy cows increased significantly, and 9 milk EV-miRNAs were differentially expressed. (4) **maternal disease:** Mirza et al. (2019) compared the milk-derived exosomal miRNAs from type 1 diabetes and healthy mothers and found that 9 immune-related miRNAs were differentially expressed. (5) **maternal lifestyle and sensitivity:** Torregrosa Paredes et al. (2014) reported that women with an anthroposophic lifestyle had lower mucin in their milk-derived exosomes. Compared with that in non-allergic mothers, the level of mucin in exosomes derived from milk supplied by allergic mothers was significantly reduced and may be associated with infant allergy. (6) **maternal stress:** Maternal lifetime stress and negative events in pregnancy were both associated with expression level of breast milk derived-EV miRNAs (Bozack et al., 2021). In addition, milk EV-miRNA expression was associated with maternal body mass index as well as and maternal smoking (Kupsco et al., 2021). Table 1 summarizes the factors influencing the expression of milk-derived EVs.

**TABLE 1:** Factors influencing the expression of milk-derived EVs.

**Factors**	**Objects**	**Outcomes**	**References**
Gestional age	Expression of exosomal peptides and miRNAs	Exosomes of preterm mothers promote the proliferation of intestinal cells better than those of term infants	Carney et al., 2017; Wang X. et al., 2019
Laction period	Concentration and contents (RNA, protein) of EVs	Immune-related miRNAs in colostrum are higher than mature milk, while tissue-specific miRNAs are more expressed in mature milk	Sun et al., 2013; Yang et al., 2017
Maternal nutrition	Concentration and contents of EVs.	Adding immunomodulator to the diet of sows changes the expression of immune-related miRNAs in sow’s milk exosomes	Sun et al., 2019
Maternal disease	Expression of EV miRNAs	9 immune-related exosomal miRNAs are differentially expressed in the milk of type 1 diabetes and healthy mothers	Mirza et al., 2019
Maternal lifestyle	Milk exosomal mucin level	Women with an anthroposophic lifestyle have lower mucin in their milk exosomes	Torregrosa Paredes et al., 2014
Maternal sensitivity	Milk exosomal mucin level	Allergic mothers have lower mucin in their milk exosomes compared with non-allergic mothers	Torregrosa Paredes et al., 2014
Maternal stress	Expression of milk EV miRNAs	Maternal lifetime stress and negative events during pregnancy are both associated with detection and expression level of human milk EV miRNAs	Bozack et al., 2021

### Stability of EVs in Breast Milk

Under physiological conditions, the intact bilayer vesicle structure of EVs can protect their internal biomolecules against degradation by ribonucleases (RNases) and digestive enzymes to maintain their integrity and biological activities (Kosaka et al., 2010b). *In vitro* studies have proved that breast milk-derived exosomes and their cargos can remain stable under conditions similar to those of the stomach or pancreas and can be further absorbed by human intestinal cells (Liao et al., 2017). Storage conditions also largely affect the concentration and integrity of milk-derived EVs. First, **temperature** is crucial. Unprocessed milk stored at −80 or 4°C induces cell death in breast milk and damage or contamination of milk-derived EVs. When human breast milk is stored at 4°C for 4 weeks, extracellular vesicles gradually decrease to 49 ± 13% of fresh breast milk (Zonneveld et al., 2014). Second, **centrifugation** should be conducted before freezing to remove fat and cell fragments, consequently reducing the number of apoptotic bodies contaminating exosomes (Zonneveld et al., 2014). Third, **ultrasonication** leads to the instantaneous destruction of the exosome membrane, with a >98% depletion of exosomal RNAs and a 20% decrease in exosome count (Leiferman et al., 2018). Fourth, microwave heating can also cause further loss of miRNAs and other cargos of milk-derived EVs (Howard et al., 2015; Zhao et al., 2018). Besides, the integrity of EVs from pasteurized milk was affected and their composition was changed (Kleinjan et al., 2021). Last, **fermentation** destroys the integrity of milk-derived exosomes *in vitro* and protein and miRNA contents in milk-derived exosomes are damaged (Yu et al., 2017).

### Physiological Functions of Breast Milk-Derived EVs in Infant Growth and Development

Both *in vivo* and *in vitro* studies have indicated that milk-derived EVs can be absorbed by mammal and exert biological activity. Milk-derived EVs supplied to mice and pigs via gavage accumulated in their intestinal mucosa, spleen, liver, heart, and brain (Zempleni et al., 2017; Manca et al., 2018; Lin et al., 2020). In addition, the pH level in the stomach of newborns is about 5; as the age of the newborn increases, the pH level decreases, and when newborns reach 2 y old, their pH is further reduced to that of an adult (pH 1–3). The acidic environment and hyperosmotic conditions of the intestines promote the stability and absorption of EVs. Liao et al. (2017) found that the milk-derived exosomes could enter human intestinal cells and localize in the nucleus, confirming that milk-derived exosomes could be absorbed by the intestinal epithelia of infants and thus perform regulatory functions. Figure 1B depicted the physiological functions of milk-derived EVs.

Current studies on the functions of breast milk-derived EVs focus on their miRNA content for the following reasons: (1) **ease of absorption:** Milk-derived EV miRNA can be absorbed by cells, penetrate the intestinal barrier, and enter the bloodstream. Milk-derived exosomes and their RNA cargos may enter the blood circulation and accumulate in various tissues of mice (Wolf et al., 2015; Kusuma et al., 2016). (2) **high expression:** Milk-derived EVs contain a large number of miRNAs. About 900 and 800 miRNAs have been detected in exosomes derived from human and bovine milk (Liao et al., 2017; Özdemir, 2020). (3) **high stability:** Compared with exogenous synthetic miRNAs, milk EV miRNAs are more resistant to harsh conditions, including repeated freeze–thaw cycles, RNA enzyme, and high temperature (Zhou et al., 2012; Liao et al., 2017; Kahn et al., 2018). (4) **strong regulatory ability:** miRNAs regulate 60% of human mRNAs and thus participate in almost every biological process (Friedman et al., 2008). (5) **high conservation:** miRNAs are highly conserved among different species, allowing them to be used across species. More than 400 miRNAs have thus far been identified in bovine milk, the majority of which have the same nucleotide sequence as that of humans. Thus, miRNAs exhibit strong potential to regulate human genes (Zhang et al., 2012; Herwijnen et al., 2018).

In this review, we summarize the effects of milk-derived EVs in three aspects: immune regulation, metabolic regulation, and neural development. Research on EV miRNA is the focus of our attention. Table 2 summarizes representative reports from the current literature on milk-derived EVs and their bioactivities.

**TABLE 2:** Summary of reports on milk-derived EVs and their bioactivities.

**Milk EVs**	**Recipient**		**Outcome**		**References**
	***In vivo***	***In vitro***	**Animal**	**Cell**	
Human	Newborn SD rat with NEC	FHC	↑The villous integrity from injury in the NEC rat model	↑Proliferation and migration of intestinal epithelial cells	Wang X. et al., 2019
Human	Newborn SD rat with NEC	IEC	↓Incidence and severity of NEC	↑Cell proliferation ↓Cell apoptosis	Pisano et al., 2020
Human	−	MDDC	−	↓HIV-1 infection ↓Viral transfer to T cells	Näslund et al., 2014
Human	−	PBMC	−	↓IL-2 and IFN-γ production ↑T regulatory cells	Admyre et al., 2007
Human	−	IEC	−	↑Cell viability ↓Oxidative stress	Martin et al., 2018
Human	−	GEC	−	↑Epithelial barrier function ↑Cell migration ↓Activation of CD4 + T cells	Zonneveld et al., 2021
Human Bovine	Mice with DSS-induced colitis	−	↓The severity of colitis ↓Histopathological scoring grade ↓IL-6 and TNF-α		Reif et al., 2020
Bovine	Mice with DSS-induced colitis		Modulate gut microbiota ↑The intestinal impermeability ↑Mucin secretion	↓Colitis-associated miRNAs, especially miR-125b	Benmoussa et al., 2019
Bovine	Mice with arthritis	−	↓The onset of arthritis ↓Cartilage pathology ↓Bone marrow inflammation	↓MCP-1 and IL-6 in serum	Arntz et al., 2015
Bovine	C57BL/6 Mice		Change microbial communities		Zhou et al., 2019
Bovine	Mice with genetic ulcerative colitis.	−	↑The subscores of stool improvement ↑Colon weight and length ↑Mucosa’s appearance		Stremmel et al., 2020
Bovine	Mdr1a−/− mice with IBD	−		Depletion of milk exosomes ↓miR-200a-3p ↓Intestinal inflammation ↓Chemokine ligand 9	Wu et al., 2019
Bovine		IEC		↓ROS level ↓Purine nucleotide catabolism ↑Energy status	Wang et al., 2021
Bovine	C57BL/6 Mice	−	Depletion of bovine milk ↓Sensorimotor gating and spatial learning		Mutai et al., 2018
Rat	−	IEC	−	↑Viability and proliferation	Hock et al., 2017
Porcine	Kunming mice	IPEC-J2	↓DON-induced damage on body weight and intestinal epithelium growth of mouse	↑Specific miRNAs and↓their targeting genes in p53 pathway ↑Cell proliferation ↓cell apoptosis	Xie et al., 2020
Porcine	Kunming mice	IPEC-J2	↓LPS-induced intestine damage and inflammation.	↓Intestinal epithelial cells apoptosis via the p53 pathway ↓Inflammation via the NF-κB pathway	Xie et al., 2019

**FHC, human normal intestinal epithelial cell line; PBMC, peripheral blood mononuclear cell; GEC, gingival epithelial cells; DSS, dextran sulfate sodium; MDDC, monocyte-derived dendritic cell; IPEC-J2, intestinal porcine enterocytes isolated from the jejunum of a neonatal unsucked piglet; DON, Deoxynivalenol; IBD, inflammatory bowel disease.**

#### Milk-Derived EVs and Immune Regulation

Breast milk is crucial in the immune system development of an infant. Owing to their abundant immunoregulatory miRNAs, milk-derived EVs may have strong immune regulatory functions. Four of the top 10 milk EV miRNAs were determined as immune-related miRNAs: miR-148a-3p, miR-30b-5p, miR-182-5p, and miR-200a-3p (Kosaka et al., 2010b). Four miRNAs (let-7a, let-7b, and let-7f, and miR-148a) were also found to have high abundance in EVs from milk collected from four species (humans, cows, pigs, pandas) examined, all of which were fully conserved at the sequence level (Herwijnen et al., 2018). Let-7a, let-7b, and let-7f were proved to regulate signal transduction by inhibiting NF-κB, suppressing immune response (Iliopoulos et al., 2009). Meanwhile, miR-148a-3p was the most highly expressed among all detected human milk-derived exosomal miRNAs, comprising 35.45% of total miRNAs (Zhou et al., 2012).

Admyre et al. (2007) found that human milk-derived exosomes could inhibit the production of inflammatory cytokines, including interleukin -2 (IL-2) and interferon (IFN), by autologous or allogeneic peripheral blood monocytes. The aforementioned exosomes also increased the number of Foxp3^+^ CD4^+^ CD25^+^ regulatory T cells, suggesting that breast milk-derived exosomes could affect the immune response of infants. Kosaka et al. (2010b) detected the expression of exosomal miRNAs in human mature milk and observed that immune-related miR-181a and miR-17 were highly expressed. Moreover, miR-181a regulated the differentiation of B cells and CD4^+^ T cells, and miR-17 regulated the development of B cells, T cells, and monocytes (Kosaka et al., 2010b). Zhou et al. (2012) reported that miR-148a-3p was highly expressed in exosomes from human milk within 6 months post-delivery, as determined by deep sequencing technology. Moreover, miR-148a was found to impair B cell tolerance by promoting the survival of immature B cells after engagement of the B cell antigen receptor, as well as inhibit the expression of the autoimmune suppressor Gadd45α, the tumor suppressor PTEN, and the pro-apoptotic protein Bim (Gonzalez-Martin et al., 2016). Näslund et al. (2014) proved that pre-exposure to human milk-derived exosomes could inhibit the infection of monocyte-derived dendritic cells with HIV-1, suggesting that milk-derived exosomes act as a novel protective factor against the vertical transmission of HIV-1. Liao et al. (2017) identified 288 distinct miRNAs in exosomes derived from human colostrum and mature milk, with hsa-miR-22-3p being the most abundant. Another study revealed that miR-22-3p significantly suppressed the activity of NF-κB, an important inflammatory signaling molecule that leads to necrotizing enterocolitis (NEC) (Takata et al., 2011). Donalisio et al. (2020) reported that human colostrum-derived EVs inhibited the viral replication of human cytomegalovirus (HCMV) and EVs surface proteins playing a role in this process. These findings help to clarify the protective mechanism of human colostrum against mother-to-child HCMV transmission.

Studies show that human milk-derived EVs can affect the intestinal immune response to bacterial attacks and protect intestines from damage (Le Doare et al., 2018). Hock et al. (2017) found that exosomes derived from mouse milk could promote the proliferation and stem cell activity of mouse intestinal epithelial cells, providing insight into the underlying mechanism underlying the positive effect of milk on NEC. Similarly, Wang X. et al. (2019) reported that human milk-derived exosomes could promote the proliferation and migration of human intestinal cells and the protective effect of human milk-derived exosomes on NEC in mouse models was confirmed in *in vivo* studies. Another study reported human breast milk-derived exosomes had a significant protective effect in intestinal epithelial cells (IEC) from oxidative stress induced by H_2_O_2_ (Martin et al., 2018). Wang et al. (2021) reported that bovine milk exosomes reduced purine nucleotide catabolism in intestinal crypt epithelial cells under H_2_O_2_-induced oxidative stress, thus exerting a protective effect against oxidative stress. In another study, Mdr1a^–/–^mice with spontaneous inflammatory bowel disease were divided into two groups: the group fed with milk exosome- and RNA-sufficient (ERS) diet and the group fed with milk exosome- and RNA-depleted (ERD) diets. The study showed that the diet lacking bovine milk-derived exosomes caused miR-200a-3p depletion and elevated intestinal inflammation in Mdr1a^–/–^mice (Wu et al., 2019). Consistent with this finding, bovine milk-derived exosomes exhibited anti-inflammatory activity in a genetic mouse model of ulcerative colitis, as reported by Stremmel et al. (2020). Li et al. (2019) supplied NEC mice with bovine milk-derived exosomes, which were found to promote the proliferation of intestinal goblet cells and inhibit the excessive intestinal inflammatory response. Meanwhile, porcine milk-derived exosomes significantly weakened the effects induced by the toxin deoxynivalenol (DON) on the body weight and intestinal epithelial growth of mice in the study by Xie et al. (2020). *In vitro* experiments demonstrated that porcine milk-derived exosomes upregulated the expression of miR-181a, miR-30c, miR-365-5p, and miR-769-3p in IPEC-J2 cells (intestinal porcine enterocytes isolated from the jejunum of neonatal unsucked piglets) but downregulated the expression of their targeting genes in the p53 pathway. These changes eventually reduced DON-induced damage by promoting cell proliferation and inhibiting cell apoptosis. Another study reported that porcine milk exosomes effectively protected the intestine epithelial cells against Lipopolysaccharide (LPS) -induced injury by protecting against apoptosis and attenuating cell inflammation via exosomal miR-iR-219, and miR-338 (Xie et al., 2019). Reif et al. (2020) reported that gavage administration of exosomes derived from bovine and human milk attenuated colitis induced by dextran sulfate sodium in murine model; in addition, it caused a significant reduction in histopathological score and the shortening of the colon, with decreases in the expression levels of interleukin 6 (IL-6) and tumor necrosis factor-α (TNF-α).

Type 1 diabetes is regarded as an autoimmune disorder. Observational and retrospective studies have determined that breastfeeding can reduce the risk of type 1 diabetes in infants (Borch-Johnsen et al., 1984), but the definite mechanism remains unclear. This protective effect may be associated with exosomes from breast milk, as suggested by Mirza et al. (2019). They detected 631 exosomal miRNAs in milk from healthy mothers and mothers with type 1 diabetes and identified 9 miRNAs that were differentially expressed in both groups. Hsa-miR-4497 and hsa-miR-178 which could increase the expression and secretion of TNF-α in human mononuclear cells, were markedly higher in exosomes derived from the milk of diabetic mothers, suggesting that the protective effect against diabetes of breastfeeding on infants could be attributed to changes in the expression of milk exosomal miRNAs.

However, relative to that in breast milk, miRNA concentration in infant formulas and their EVs is markedly low or might not even be detected (Chen et al., 2010; Golan-Gerstl et al., 2017; Leiferman et al., 2019), which may partly explain the benefits of breastfeeding to infants. Incorporating immunomodulatory milk-derived EVs into infant formula milk powder may provide a new option for the prevention of infants’ immune disorders.

#### Milk-Derived EVs and Metabolic Regulation

EVs mediate communication among metabolic organs and tissues, including islet cells, the liver, adipose tissue, and skeletal muscle (Guay and Regazzi, 2017). Afrisham et al. (2020) found that the plasma exosomes derived from obese patients could increase triglyceride synthesis and impair insulin signal transmission in hepatic cells. Adipose tissue-derived EVs from obese mice caused glucose intolerance and insulin resistance in mice of normal weight (Deng et al., 2009), while adipose tissue-derived EVs from lean mice could improve glucose tolerance and insulin sensitivity when administered to obese mice (Ying et al., 2017). Brown adipose tissue (BAT) which can promote energy consumption have shown great potential in obesity treatment. Zhou et al. (2020) reported that BAT-derived exosomes alleviated the metabolic syndrome in high-fat-diet induced obese mice. Furthermore, this protective effect of BAT- derived exosomes might be related to exosomal proteins involved in mitochondria functions (Zhou et al., 2020). In the experiment conducted by Kumar et al. (2021), exosomes were isolated from the feces of obese mice fed with a high-fat diet. These exosomes were given to lean mice, which then exhibited resistance to insulin. This resistance could be attributed to the lipid composition of exosomes. Besides proteins and lipids, exosomal miRNAs, play a vital role in metabolic regulation. In the study conducted by Ying et al. (2017), they determined that these metabolic regulatory functions of adipose tissue-derived exosomes were mainly associated with the differential expression of its content: miR-155. Pancreatic beta cells derived exosomes alleviates type 2 diabetes by improving peripheral insulin sensitivity and preserving β cell function via exosomal miR-26a (Xu et al., 2020). Another study found that M2-polarized macrophages derived exosomes could improve insulin sensitivity in obese mice via exosomal miR-690 (Ying et al., 2021).

Studies show that breast milk-derived EVs are rich in various miRNAs (such as miR-148a, miR-92a, miR-146a, miR-125b) which exert regulatory effects on metabolic regulation (Zeng et al., 2021). Our previous studies found that the ectopic expression of miR-148a could accelerate differentiation and partly rescue the inhibition of WNT1-mediated lipogenesis (Shi et al., 2015). miR-92a protected pancreatic β cells function by targeting KLF2 in diabetes (Wang et al., 2018). miR-146a inhibited adipogenensis through TGF-β and AKT/mTORC1 signal pathways by targeting SMAD4 and TRAF6 in porcine intramuscular preadipocytes (Zhang et al., 2021). miR-125b-5p could suppresses atherosclerotic plaque formation via inhibiting Map4k4 (Lin et al., 2021).

On the basis of the effects of EVs from other sources on metabolic regulation and miRNAs associated with metabolism in milk-derived EVs, we hypothesized that milk-derived exosomes have similar metabolic regulatory functions. However, few studies have been thus far been reported on the role of breast milk-derived EVs in metabolic regulation. As the most important nutritional source during infancy, breast milk is a protective factor for metabolic disease (Victora et al., 2016; Souza et al., 2020). The mechanism underlying the long-term health effects of breast milk has yet to be clarified, which may be related to the “metabolic imprinting” of bioactive factors such as EVs in milk. In general, more studies need to be conducted to elucidate the effects of breast milk-derived EVs on metabolic regulation.

#### Milk-Derived EVs and Neural Development

With advances in EVs research, the effect of milk-derived EVs on neural development has been determined. Researchers detected miRNAs that positively influenced synaptic development in mammals, half of which were found in the top 288 miRNAs expressed in human milk-derived exosomes (Lonnerdal, 2019). This finding suggested that neurodevelopment in early life could benefit from these bioactive factors. For instance, let-7a/b is involved in promoting neuronal differentiation (Bian et al., 2013). After ingestion by mice, milk-derived exosomes and exosomal miRNAs accumulated in the brain, suggesting that milk-derived exosomes could exert bioactivities in neurodevelopment (Manca et al., 2018). Purinergic signaling in the brain plays an important role in spatial learning and memory (Burnstock et al., 2011). Unlike the mouse fed with an ERS diet, the mouse fed with an ERD diet exhibited a metabolic abnormality of purine and significant loss of spatial learning and auditory startle response in mice (Mutai et al., 2018). Similarly, the concentration of urinary purine metabolites was significantly higher in infants fed with soy milk or formula than breast-fed infants. This difference suggested that the absence of breast milk-derived exosomes could affect normal brain function in infants (Aguilar-Lozano et al., 2018). A longitudinal study indicated that psychomotor development is higher in breastfeeding babies than in babies fed with formula milk or soy milk. Breastfeeding was positively correlated with the mental development index of children aged 6 and 12 months; moreover, the index of breastfed babies was significantly higher than that of babies fed with formula or soy milk (Andres et al., 2013). The mechanism underlying the effect of breast milk on neurodevelopment in infants has yet to be clarified, but milk-derived EVs may partly play a role in it.

### Potential Application of Milk-Derived EVs

The potential of EVs as therapeutic drugs is actively being explored. Milk-derived EVs have been proved to be a novel and effective therapeutic for NEC, a serious disease that threatens life of premature infants. Using NEC animal models, studies have demonstrated that human milk-derived EVs had anti-apoptotic and pro-proliferative effects, reducing the incidence and alleviating the symptoms of NEC (Wang X. et al., 2019; Pisano et al., 2020). Arntz et al. (2015) found that bovine milk-derived exosomes could alleviate arthritis in mice. In the experiment conducted by Yun et al. (2020), the osteoporotic mice that were orally administered with exosomes for 2 months significantly improved their bone mineral density relative to that of the control group (i.e., without exosome treatment), suggesting the protective effect of milk-derived exosomes against osteoporosis *in vivo*. The etiology of multiple sclerosis (MS) involves the deterioration of myelin-oligodendrocyte glycoprotein (MOG) in the myelin sheath of neurons, leading to abnormal nerve impulse transmission. The surface of milk-derived EVs contains casein protein, which exhibits a 50% sequence homology with that of MOG. Studies have shown that milk-derived EVs can treat MS in mouse models (Mokarizadeh et al., 2015); in addition, the application of milk-derived EVs in the treatment of MS in humans requires further study.

Milk EVs can also provide biocompatible vehicles for the delivery of drugs, allowing medical nutritionists and clinicians to develop safe and targeted therapies for the treatment of various pathologies (Adriano et al., 2021). Somiya et al. (2018) isolated purer EVs from bovine milk and the EVs showed no systemic toxicity and immunogenicity in mice. The pH of the stomach is about 1–3 in normal adults. Lactoferrin extracted from breast milk is degraded to a pH of 2; however, when loaded into exosomes, lactoferrin is protected from proteolysis and then absorbed by intestinal cells (Lönnerdal et al., 2011). In other studies, the use of milk-derived exosomes to deliver siRNA targeting cancer genes has achieved good efficacy (Aqil et al., 2019; Munagala et al., 2021). Gene regulators delivered via breast milk-derived EVs generally present numerous advantages. They exhibit cross-species biocompatibility and improved circulating half-lives, cross hematoencephalic and placental barriers, and are intravenously administered with few side effects (Munagala et al., 2016; Del Pozo-Acebo et al., 2021; Grossen et al., 2021). Robust studies are needed to further explore the potential application of milk-derived EVs as therapeutic agents and drug carriers.

## Conclusion and Prospect

EVs, which differ in the content of cell original proteins, lipids, and nucleic acids they deliver, provide cell–cell communication and are largely involved in various physiological and pathological processes in the body. These processes include immune and metabolic regulation, the development of tumor, neuromuscular disease, and other conditions. Further research elucidates the mechanism by which EVs are generated, secreted, absorbed, and are allowed to exert important biological effects. As the most critical nutritional source in the early stages of human life, breast milk affects infant health and even adult health. Breast milk-derived EVs are rich in miRNAs, which are closely associated with immune regulation, metabolic regulation, and neural development in infants. Milk-derived EVs have recently been explored as a tool for mother-child information transmission, although the underlying mechanism remains poorly understood. Deep studies on the biological properties, activities, and significances of milk-derived EVs should be encouraged. Existing research on milk-derived EVs has the following deficiencies and gaps: (1) First, most studies are focused on animals, such as bovine milk or porcine milk, and more reports on human breast milk are needed in the future to reveal the important role of human milk-derived EVs in infant growth and development. (2) Second, miRNAs are highly conserved across different species; thus, research on milk-derived EV contents focuses on miRNA. Conversely, other RNAs, such as lncRNA and circRNA, are less conservative than miRNA; moreover, their functions are mostly unknown, impeding the study of the biological functions of these EV RNAs. The functions of EV proteins and lipids, in addition to milk EV RNAs, are also poorly understood. (3) With regard to the future application of milk-derived EVs, proper isolation and storage of exosomes is necessary to maintain their biological activity. Adding physiological levels of milk-derived EVs into infant formula may prevent certain neonatal diseases such as NEC in formula-fed infants. In addition, the potential of milk-derived EVs as therapeutic agents and drug carriers also deserves further investigation.

## Author Contributions

XJ and LY wrote the original draft preparation. ZZ and XCu constructed the main conceptual ideas and outlined the proof. HZ and XS gathered and sorted the literatures. CJ and XCh provided funding and approved the proof. All authors have read and agreed to the published version of the manuscript.

## Conflict of Interest

The authors declare that the research was conducted in the absence of any commercial or financial relationships that could be construed as a potential conflict of interest.

## Footnotes

1 http://www.exocarta.org/
